# Microwave-Assisted Preparation of Luminescent Inorganic Materials: A Fast Route to Light Conversion and Storage Phosphors

**DOI:** 10.3390/molecules26102882

**Published:** 2021-05-13

**Authors:** José Miranda de Carvalho, Cássio Cardoso Santos Pedroso, Matheus Salgado de Nichile Saula, Maria Claudia França Cunha Felinto, Hermi Felinto de Brito

**Affiliations:** 1 Institute of Physics, University of São Paulo, São Paulo BR-05508-900, SP, Brazil; 2 The Molecular Foundry, Lawrence Berkeley National Laboratory, Berkeley, CA 94720, USA; ccspedroso@lbl.gov; 3 Institute of Chemistry, University of São Paulo, São Paulo BR-05508-000, SP, Brazil; matheus.saula@usp.br (M.S.d.N.S.); hefbrito@iq.usp.br (H.F.d.B.); 4 Nuclear and Energy Research Institute, São Paulo BR-05508-000, SP, Brazil; mfelinto@ipen.br

**Keywords:** microwave-assisted synthesis, luminescent inorganic materials, light converting, energy storage, microwave heating

## Abstract

Luminescent inorganic materials are used in several technological applications such as light-emitting displays, white LEDs for illumination, bioimaging, and photodynamic therapy. Usually, inorganic phosphors (e.g., complex oxides, silicates) need high temperatures and, in some cases, specific atmospheres to be formed or to obtain a homogeneous composition. Low ionic diffusion and high melting points of the precursors lead to long processing times in these solid-state syntheses with a cost in energy consumption when conventional heating methods are applied. Microwave-assisted synthesis relies on selective, volumetric heating attributed to the electromagnetic radiation interaction with the matter. The microwave heating allows for rapid heating rates and small temperature gradients yielding homogeneous, well-formed materials swiftly. Luminescent inorganic materials can benefit significantly from the microwave-assisted synthesis for high homogeneity, diverse morphology, and rapid screening of different compositions. The rapid screening allows for fast material investigation, whereas the benefits of enhanced homogeneity include improvement in the optical properties such as quantum yields and storage capacity.

## 1. Introduction

### 1.1. Luminescent Materials: Optical Properties and Applications 

Luminescent materials, also known as phosphors, usually refers to inorganic materials that emit light after external physical stimuli, such as UV-VIS, IR-Lasers, X-rays, γ-rays, and e-beams [1]. These materials are composed of a host lattice with intentionally added optically active impurities (dopants). The dopants act as luminescent centers, converting the incident excitation into a characteristic light emission that depends on the dopant’s identity [2]. The most common dopants for luminescent materials are the f-metals, such as the trivalent rare-earths (RE^3+^: Pr, Nd, Eu, Tb, Dy, Er), that emit light after optical transitions within the f-electron manifold [2]. The f-electrons are strongly shielded from the chemical environment and retain atomic characteristics, yielding sharp emission lines with wavelengths nearly independent of the host’s composition. The f-f electronic transitions are parity forbidden and, thus, the emission decay times are long (μs-ms). 

**Figure 1:**
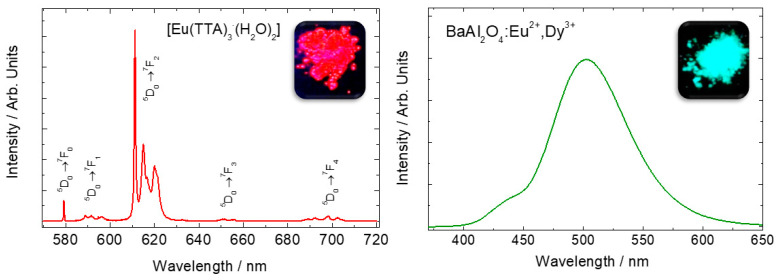
Emission spectra of Eu^3+^ in Eu(TTA)_3_·(H_2_O)_2_ complex (**left**), and Eu^2+^-doped BaAl_2_O_4_ host (**right**). Eu^3+^ sharp lines spectra is attributed to f-f transitions whereas the Eu^2+^ broad emission band is attributed to f-d transitions. Adapted from references [3,4]. Copyright © 1997 Published by Elsevier B.V.

Some rare earth ions (e.g., Ce^3+^, and Eu^2+^) behave as crystal-field sensitive ions, having intense broad emission bands with emission color dependent on the host’s composition. In these cases, the emission is governed by parity-allowed 4*f*-5*d* transitions, yielding intense emission bands with fast decay times (ns-μs). Figure 1 provides the comparison between the *f-f* and *f-d* transitions for the Eu^3+^ and Eu^2+^ ions. 

Various transition metals also act as luminescent centers, e.g., Mn^4+^-doped K_2_SiF_6_ [5], and Cr^3+^-doped ZnGa_2_O_4_ [6]. In these cases, parity-forbidden d-d electronic transition is responsible for the emission. The d-orbitals broadly interact with the chemical environment leading to strong crystal field splitting according to the composition of the materials. Thus, most of the transition metals’ absorption and emission bands are broad. However, sharp emissions can also be observed, such as Mn^4+^-doped K_3_ScF_6_ red phosphors [7]. 

**Figure 2:**
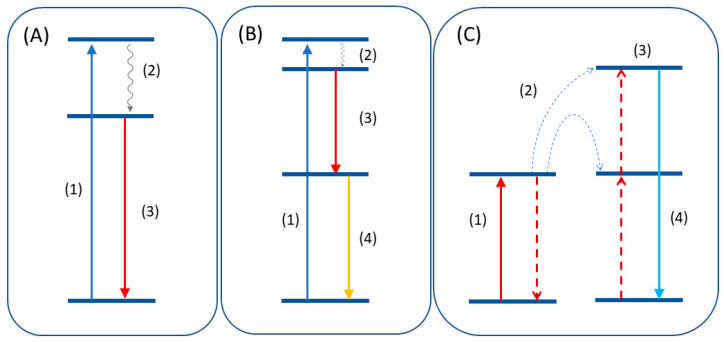
(**A**) Downshift mechanism consisting of excitation (1), non-radiative decay (2), and emission (3). (**B**) Quantum cutting mechanism where the emission of two photons (3,4) occurs after the excitation. (**C**) Upconversion emission after energy transfer (2) of two lower energy quanta to a higher excited state (3).

Luminescent materials can act as light converters due to different photophysical processes, such as downconversion (or quantum cutting) [8,9,10], upconversion [11,12,13], and down-shifting [14,15] (Figure 2A–C). Downshifting is the most conventional photoluminescence mechanism, where high-energy photons are converted into low-energy photons. Ideally, all excited photons are converted to emitted photons without losses in the optical process, obtaining a quantum yield of 100% [16,17,18,19]. On the other hand, the first experimental evidence for quantum yields higher than 100% was reported for YF_3_:Pr^3+^ material [17,20]. This optical phenomenon proposed by Dexter consists of the high-energy photons splitting into two or more lower-energy photons. This mechanism, called downconversion or quantum cutting, involves single or multiple ions (e.g., in YF_3_:Pr^3+^ [17,20] or YPO_4_:Yb^3+^, Tb^3+^ [10], respectively).

Upconversion (UC) is a nonlinear optical phenomenon that converts two or more low-energy photons to a higher-energy photon [13]. This optical process, known as anti-Stokes emission, was theoretically proposed by the physicist Nicolaas Bloembergen in 1959 [21]. The first experimental result, reported by François Auzel in 1966, consisted of the energy transfer from Yb^3+^ to Er^3+^ and Tm^3+^ ions [13]. The UC process has since inspired light conversion mainly from near-infrared (NIR) to UV-visible-NIR light, suitable for bioimaging, solar cells, anticounterfeiting, photocatalysis, and photodynamic therapy [22].

Persistent luminescence (PeL) materials continue to emit light after removing the excitation source due to stored energy in the structural defects that act as charge carrier traps [23,24,25]. After the removal of the excitation, the material absorbs available thermal (kT) or optical (hv) energy to release the trapped charge carriers that recombine with the emitting centers. The radiative decay of the excited states emits a photon with a characteristic wavelength. The most efficient PeL phosphors are dominated by Eu^2+^-doped inorganic materials, exhibiting 24 to 48 h of persistent emission after few minutes of excitation in the UV-VIS range [25]. However, plenty of examples for d- and f-metals are available, and extensive reviews on the topic have been published [23,24]. 

Luminescent materials have several applications in technology, telecommunications, and health sciences, and are the subject of extensive research. Specific applications can be in light-emitting diodes [26,27,28], laser [29], thermometry [30], thermoluminescence dating [31,32], dosimetry [33], bioimaging [6,34], and photodynamic therapy [35,36,37]. 

For instance, phosphor converters for LEDs (pc-LEDs) work as light converters where an excitation source, e.g., blue emitting-LEDs, excites the luminescent material that emits broadband in the yellow region. This kind of phosphor has defined characteristics, such as high quantum yields and fast excited-state decay times. The most efficient light converters for pc-LED are inorganic hosts doped mainly with Ce^3+^ and Eu^2+^ [27,38,39,40]. The parity-allowed (f–d) electronic transition in Ce^3+^ and Eu^2+^ ions results in high-intensity emissions with short excited-state lifetimes (μs-ns). However, other examples can be easily found in trivalent rare earths, Eu^3+^, Tb^3+^, Pr^3+^, Dy^3+^, and *d*-metals (Mn^4+^) doped materials [5,7,41,42,43,44]. PeL materials are in evidence nowadays due to vast and unexplored bioimaging applications [37], theranostics [45], and photocatalysis [46]. When it comes to application, persistent phosphors must have high quantum yield and high storage capacity [47,48].

### 1.2. Concepts and Mechanisms of Microwave Heating

Microwaves (MW) are electromagnetic radiation in the frequency interval of 300 MHz (1.24 μeV; 1 m) to 300 GHz (1.24 meV; 1 mm). Radiation in this range is mainly used in wireless telecommunication protocols, such as Bluetooth and wi-fi modems [49]. A narrow part of the microwave spectrum is used for industrial, domestic, scientific, and medical purposes (ISM bands) [50]. The industrial and domestic uses of microwaves come from the ability to heat certain materials depending on their dielectric properties [49,51]. For instance, the radiation in a domestic microwave oven (2.45 GHz; λ: 12.2 cm) interacts with the water contained in the food, heating it swiftly due to the intrinsic dielectric properties of the water molecules. Further, a precise tuning of microwave radiation parameters enables microwave heating of different materials, such as metal oxides [52,53], carbon [49,54,55], and organic solvents [56]. 

**Figure 3:**
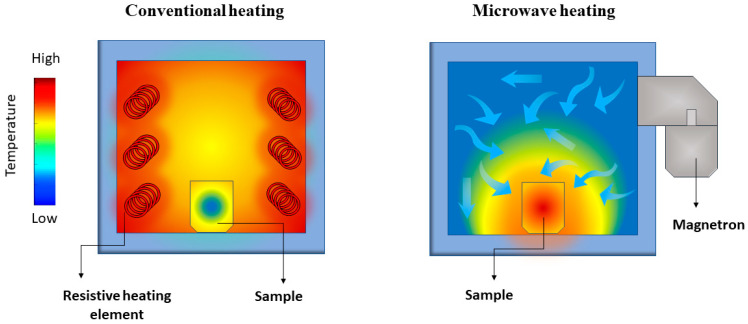
Representation of the temperature gradient in conventional (**left**) and microwave-assisted (**right**) heating of a solid sample.

Microwave heating properties differ significantly from conventional heating ones. In conventional heating, the external source, such as a resistive furnace, heats the material’s surface, leading to a large temperature gradient compared to the material’s core. Instead, dielectric heating occurs volumetrically, meaning that the material irradiated by the microwaves is heated locally, leading to a small temperature gradient [56] (Figure 3). Microwave-assisted heating rates are drastically steeper than conventional heating because of the absence of heat transfer from an external source.

The combination of these properties can be highly beneficial to the synthesis of inorganic materials using microwave-assisted approaches. For instance, solid-state methods benefit significantly from dielectric heating, shortening the synthesis time from days and hours to tens of minutes. In addition, it can yield more uniform, homogeneous materials [57].

The principles of microwave heating are based on the material’s response to the electromagnetic fields’ alternating nature. Bhattacharya et al. [49] discussed in detail the microwave heating mechanism in several materials. The relevant parameters for the heating include the dielectric constant (ε′) and dielectric loss (ε′′). The parameters can be interpreted as the ability of the material to store (ε′) and convert (ε′′) the electric energy from the microwave irradiation.

The main mechanism of dielectric heating is attributed to the polarization losses that comprise dipolar, interfacial, ionic, and electronic losses [49,51]. All these contributions are related to the intrinsic properties of the materials. Thus, a corrected assessment of the microwave interaction with a particular material must include a detailed analysis of its dielectric properties. Additionally, if the material to be irradiated has high conductivity, then the conduction loss enters the summation in the dielectric loss constant (ε′′). Magnetic contributions must be considered when the material presents a magnetic moment. Most luminescent materials are not intrinsically magnetic, making it possible to simplify the equation to only the electric contribution.

The interaction of microwaves with matter can be understood as how much a microwave can penetrate in a specific material. The penetration depth of a microwave within materials interacting only with the electric field (μ″_r_ = 0) can be expressed by Equation (1):

(1) $$dp=\frac{c}{\sqrt{2\pi f}\sqrt{\varepsilon \prime_{r}\mu \prime_{r}}}{\left[\left(\sqrt{1+\tan^{2}\delta_{e}}\right)-1\right]}^{-1/2}$$

where *f* is the frequency of the microwave, (ε′_r_) is the relative dielectric constant, and (ε′′_r_) is the relative dielectric loss. The loss tangent ($\tan\delta_{e})$ is an important parameter that denotes the dissipation of the electrical energy quantitatively due to multiple physical processes [58], and can be defined by Equation (2):

(2) $$\tan\delta_{e}=\frac{\varepsilon_{r}^{\prime \prime }}{\varepsilon_{r}^{\prime }}$$

The *dp* parameter will then classify the materials’ interaction with the microwave in three different classes, (i) opaque or reflector, (ii) transparent, and (iii) absorber [49]. If the penetration depth is shallow (micrometer range), the microwave is reflected by the material’s surface. Typical examples of microwave reflectors are metals like Cu (*dp* = 1.3 μm) and Al (*dp* = 1.7 μm). Alternatively, if the penetration depth is too large, then the microwave goes through the material without any interaction being transmitted. Materials like alumina, borosilicate glasses, and zirconia are excellent examples of microwave-transparent materials, with penetration depths larger than 10 m.

Neither of these situations is ideal for heating the materials and therefore performing microwave-assisted synthesis. To interact with microwaves, the radiation’s penetration depth must be optimal to dissipate the electrical energy efficiently. Absorber materials have penetration depths in the order of centimeters, such as SiC (*dp* = 1.9 cm), water (3.0 cm), and carbon black (5.7 cm) [59,60,61]. The tangent loss of the materials in terms of reflector, transparent and absorber have typical values of $\tan\delta_{e}\;$ < 0.01 (transparent), >0.1 (absorbers), and >>1 (reflectors).

The fact that the dielectric properties of the materials can vary with temperature increases possibilities of interacting with materials by changing their reactional medium temperature. For instance, yttria-stabilized zirconia is microwave-transparent up to 200 °C ($\tan\delta_{e}=0.001$; dp=5.68 m), suffering a transition to an absorber material at 600 °C ($\tan\delta_{e}=0.15$; dp=0.039 m). The principle of intrinsic tangent loss modulated by the materials’ temperature is beneficial for synthetic purposes. A synthetic precursor consisting of a mixture of low-lossy materials (transparent) can be in close contact with a high-lossy (absorber) material that will initially interact with the microwave, generating local heating. The heat transfer to the low-lossy material can inflict a change in the tangent loss, transitioning from transparent to absorber. This type of heating can be denominated as hybrid microwave heating, and it is the most-used method in microwave-assisted solid-state (MASS) synthesis [49,51,62,63].

### 1.3. Properties of Microwave Heating

Microwave-assisted heating can be characterized by a combination of unique properties offering faster and more efficient heating, often leading to more crystalline, homogeneous materials [64]. Firstly, the direct energy conversion into heat leads to a smaller temperature gradient throughout the precursors. In conventional resistive heating, the large temperature gradients make the reactions sluggish, taking a long time to reach equilibrium and generate the products. Dielectric heating works in the opposite manner, heating the materials from the inside to the outside. Dielectric heating leads to minimal energy consumption and faster reaction rates [65]. The thermal efficiency is an appealing point when considering laboratory work for sample screening and compositional tailoring. High throughput can be obtained with specialized setups, allowing the synthesis of tens of samples in few minutes. The fast screening in compositional tailoring can be beneficial for luminescent materials with a strong correlation of the optical property with the composition and structure.

Dielectric heating is also volumetric by nature, meaning that the heating is uniform throughout the sample, in theory. In actual samples, the volumetric heating is not perfect due to inhomogeneity in the precursors and temperature dependence of the dielectric properties [66]. However, compared with conventional heating, the MASS method has more uniform heating and, therefore, a more homogeneous composition of the final products. For luminescent materials, this property is of utmost importance once the homogeneous dispersion of activator and sensitizer ions in the material’s host leads to higher quantum yields and increased brightness [67]. Homogeneous luminescent materials offer a more-defined local structure that can lead to a precise control of the emitting color of crystal field-sensitive ions, such as Eu^2+^ [57].

**Figure 4:**
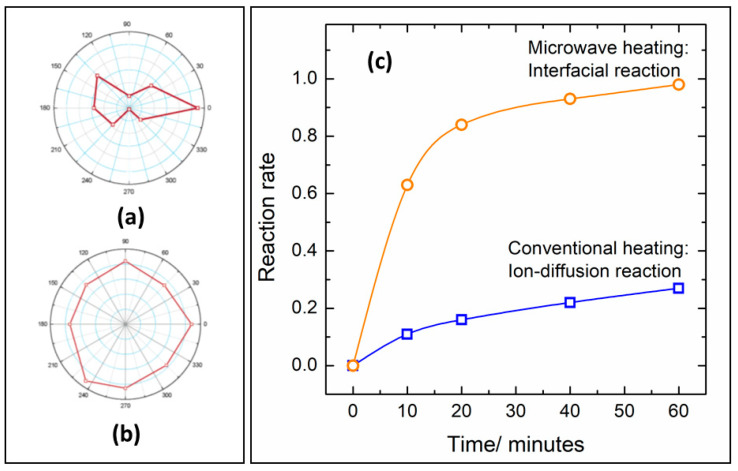
Ionic diffusion in a ceramic heated by polarized microwave irradiation (**a**) and conventional heating (**b**). Adapted from reference [70]. Reaction rates of NiFe_2_O_4_ synthesis under microwave heating (orange) and resistive heating (blue) at 850 °C (**c**). Adapted from ref. [71]. Copyright © 2005, American Chemical Society.

The ionic diffusion limit encountered in conventional heating methods is also a significant problem in solid-state synthesis [64,68]. Usually, the diffusion is exceptionally slow if the temperature of the synthesis is lower than two thirds of the precursor’s melting point (Tamann’s rule) [69], which translates to temperatures usually higher than 1000 °C. The microwave radiation effect on ionic diffusion in bulk inorganic materials was investigated by Whittaker et al. [70]. The use of polarized MW irradiation combined with x-ray fluorescence technique has shown a preferential ionic migration in the polarized axis of the radiation (Figure 4a–b).

The increased diffusion is related to increased reaction rates in microwave-heated synthesis in comparison with resistive furnaces. Vanetsev et al. [71]. studied the kinetics of NiFe_2_O_4_ formation by conventional and microwave-assisted solid-state reaction. Besides the faster reaction rates, microwave irradiation also promotes changes in the rate-controlling stage of the synthesis. By removing the diffusion hindrances due to non-thermal ponderomotive forces, the rate-controlling step of the synthesis changes from ion-diffusion to the interfacial chemical reaction step (Figure 4c) [71].

Instantaneous heating is also an essential feature of dielectric heating, swiftly converting the MW energy into heat. Correspondingly, once the microwave irradiation is ceased, the heating stops immediately, causing the reaction to quench instantaneously. This could lead to metastable phases, otherwise impossible to synthesize using conventional methods. The temperature quenching can be greatly beneficial for persistent luminescence phosphors because of the defect structure caused by rapid heating and cooling rates.

Once the dielectric constant and tangent loss vary with the material composition and structure, microwave irradiation allows for selective heating of the reactants [64]. This effect can find incredible applications on heating specific sites in supported metal catalysts [72]. The synthesis of chalcogenides greatly benefits the selective heating, allowing the reaction to occur before the sulfur evaporation [73].

**Figure 5:**
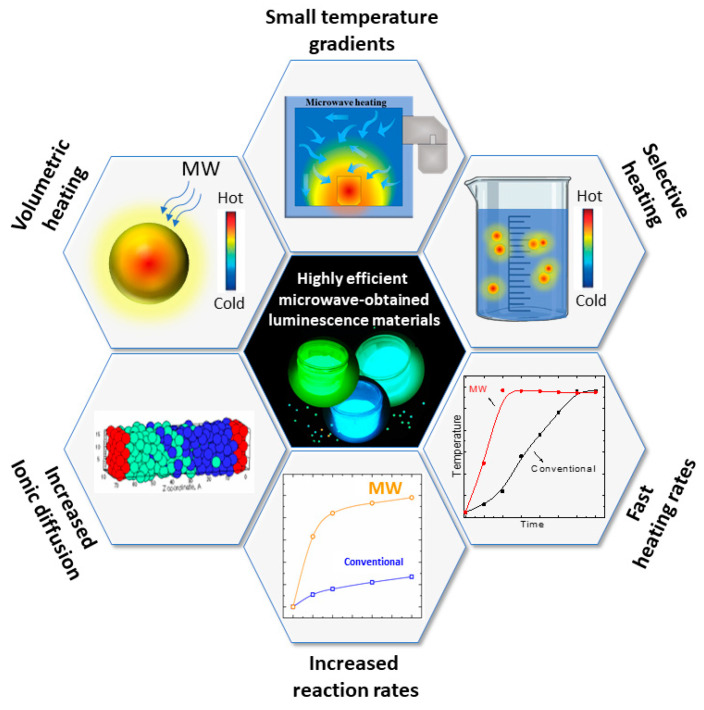
Fundamental microwave-assisted heating properties that are beneficial in preparing luminescent materials.

The properties of microwave-assisted heating are summarized in Figure 5. The combination of features allows for the synthesis of highly efficient luminescent materials with singular properties, as will be discussed in detail in the next sections.

## 2. Methodologies of Microwave-Assisted Synthesis

There are several approaches to obtaining luminescent materials using microwave-assisted methodologies. However, microwave-assisted syntheses needs a detailed design of the experimental apparatus due to many dielectric heating parameters. The synthesis setup needs to be modified depending on the application to achieve optimum performance and reproducible results. Here, we present some details on the most common methods to obtain solid inorganic luminescent materials using microwave-assisted heating. Each one has advantages and disadvantages, and the materials’ application should be considered when choosing the method as will be discussed further in next sections.

### 2.1. Microwave-Assisted Solid-State (MASS) Synthesis

Although there are some commercial setups for MASS synthesis, most of the literature relies on the usage of a domestic microwave oven (DMO) [49,54,55,63,74,75]. This is probably because of two main reasons: (i) economic viability: DMOs are inexpensive equipment, and (ii) DMOs are easily customizable to meet the needs of many methods.

The usage of DMOs comes with the price of several safety issues that need to be addressed carefully. In a customized DMO setup, the researcher needs to avoid radiation leakage, harmful to human body tissues at the frequency of 2.45 GHz. Additionally, DMO setups should be placed in a fume hood to avoid inhalation of toxic gasses from reactions. Effective thermal insulation of the reactional vessel is indispensable to prevent damage to the microwave oven.

To achieve optimum reproducibility, one needs to be consistent with the parameters of the synthesis, with a carefully chosen irradiation program. Crucial synthesis parameters are the crucible sizes, amount of starting materials, nature and mass of the susceptor, position inside the microwave, radiation frequency, and irradiation power levels [63].

**Figure 6:**
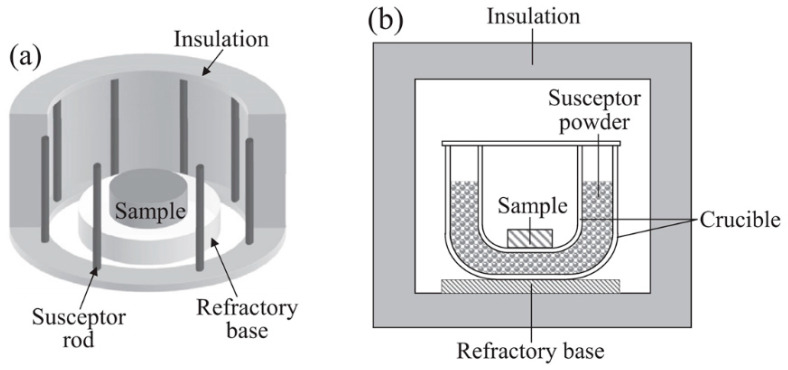
(**a**) Microwave-assisted solid-state synthesis setup with hybrid heating using SiC rods as susceptors. (**b**) MASS synthesis setup using a DMO. Susceptor surrounding the sample absorbs the microwave and initiate the heating [49]. Copyright © 2016, with permission from Elsevier.

A typical setup for microwave-assisted solid-state synthesis is depicted in Figure 6a,b. The setup for MASS (Figure 6b) synthesis consists of three parts [76,77]:

(i) Reaction vessel: crucible where the precursor powder will be placed, and the reaction occurs. Usually, the crucibles are made of alumina (Al_2_O_3_) due to its high thermal and chemical stability. For specific purposes other crucibles can be used, such as zirconia or graphite. The ideal size of the crucibles is up for debate, but crucibles varying from 3 to 10 mL in volume are the most common.

(ii) Susceptor: material that absorbs 2.45 GHz microwaves efficiently at room temperature. Due to the fixed frequency of the radiation in DMOs, few oxides directly interact with the microwave. Since a significant number of luminescent materials use oxides as starting materials, most of the works in literature use the hybrid heating approach, where a susceptor (e.g., carbon) is used to initiate the heating (Table 1). The susceptor also has a secondary effect of generating a local atmosphere. The composition of the local atmosphere must be considered when synthesizing the materials. For example, a mildly reducing atmosphere is obtained when using carbon as a susceptor due to its incomplete burn that generates CO gas.

(iii) Thermal insulation: Insulating blocks where the reaction and susceptor crucibles will be placed to insulate extreme temperatures of the solid-state synthesis. Usually aluminosilicate bricks are used to host the reaction system (precursors + susceptors). Alumina foam and glass wool are also reported in the literature. The criteria to choose the correct system is the maximum thermal insulation allied to the high MW transparency.

The three-part system is inserted in a DMO and irradiated using time vs. power level program usually defined by measuring the temperature increase rate. Although temperature measurement is not an easy task, it is of utmost importance to avoid underreacted samples or decomposition by excessive temperatures [76]. A pyrometer is a convenient tool to measure the instantaneous temperature of the crucible. However, the recorded temperature is just of the material’s surface. The temperature inside the susceptor should be considerably higher because the microwave penetration promotes heating initially below the surface of the material [78]. The same effect can be expected for the sample, where the core is hotter than the surface. Despite the difference in temperature between the core and surface, the use of hybrid heating using a susceptor helps obtain a more homogeneous heating of the samples compared to direct heating [78].

**Figure 7:**
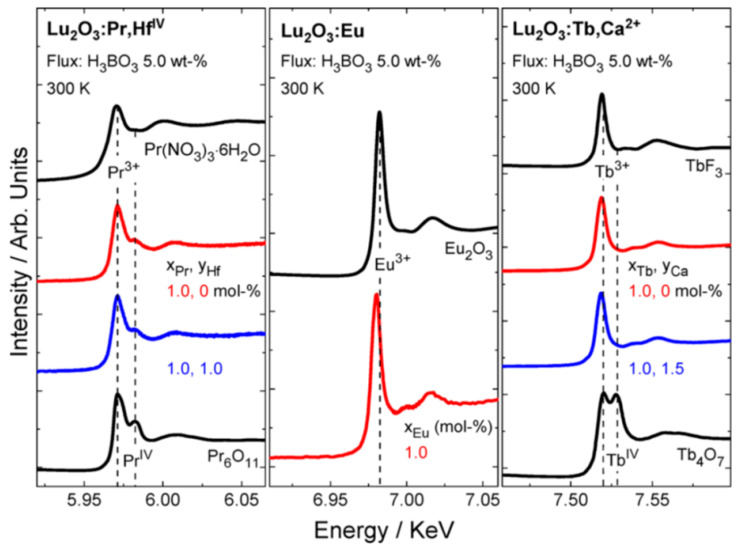
L_III_ edge XANES spectra of Pr (**left**), Eu (**middle**), and Tb (**right**) doped Lu_2_O_3_. Spectra for reference materials are also provided [62]. Copyright © 2016, American Chemical Society.

Carbon is the most-used susceptor and can absorb microwave radiation from DMOs reaching rapidly high temperatures (Table 1). Additionally, carbon undergoes through an incomplete burn at high temperatures, generating CO reducing gas. The local, reducing atmosphere can be beneficial if the luminescent material to be prepared needs to reduce activator ions’ initial oxidation state. For instance, Eu^2+^ doped in Sr_2_MgSi_2_O_7_ phosphors, which need to be reduced from the trivalent state in the precursor, e.g., Eu_2_O_3_ [57]. Tb_4_O_7_ is a common precursor for Tb-doped phosphors prepared by the solid-state method, sometimes needing H_2_ gas to be reduced from Tb^4+^ to Tb^3+^ [82]. With the MASS method, it is possible to achieve Lu_2_O_3_:Tb^3+^ in a pure trivalent state, in 20 min, using activated carbon as susceptor (Figure 7) [62]. When preparing Y_3_Al_5_O_12_:Ce^3+^, the pure tetravalent form of cerium oxide, CeO_2_, is reduced to Ce^3+^ during the MASS synthesis in a single step of ca. 20 min [83].

Silicon carbide, SiC, is also a good option for susceptors because of its high-temperature stability (2830 °C). SiC is common in commercial microwave ashing furnaces that use SiC plates or rods to convert the MW radiation into heat (Figure 6a) [84]. Despite a slow initial heating rate, SiC has a monotonic increase of temperature, from 25 to 600 °C, working as a steady microwave heating element. After 600 °C, the microwave heating rate increases dramatically, reaching 1800 °C in few minutes [80]. CuO has some usage as a reactant‑susceptor, used as a reactant in the MASS synthesis of YBa_2_Cu_3_O_7−x_ superconductors [53].

### 2.2. Microwave-Assisted Combustion (MAC)

Combustion relies on rapid oxidation that generates heat or as a sequence of exothermic chemical reactions between a fuel and an oxidant that is accompanied by the production of heat and the conversion of chemical species [85,86,87,88]. In the volume reaction model, also referred to as thermal explosion, the entire sample is uniformly heated to the ignition temperature, and the combustion reaction coincides in all parts of the sample.

The conventional combustion synthesis uses a certain amount of oxidizer (e.g., metal nitrates) and fuel (e.g., urea) to produce an aqueous solution that, upon heat, ignites the combustion [87,88]. The microwave-assisted method works in the same way, but the formed solution needs to absorb the microwave radiation to initiate the heat. Several solvents (Table 2) have a suitable dielectric tangent loss to interact with the microwave electric field and convert it into heat.

**Figure 8:**
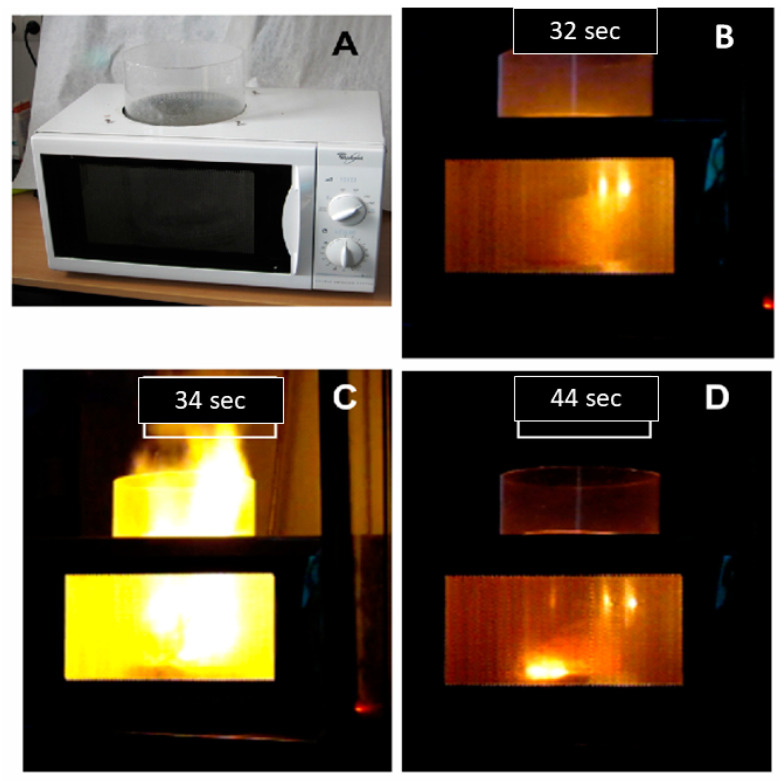
Microwave-assisted combustion synthesis setup using DMO equipment (**A**). Synthesis progression at 32 (**B**), 34 (**C**), and 44 s (**D**). Such extensive modification in DMOs needs thorough consideration of the involved hazards, mainly microwave leakage. Reproduced from ref. [85,90] with permission from The Royal Society of Chemistry.

The combustion synthesis has many advantages such as high reaction temperatures with relatively low preheating temperatures, high heating rates, short duration of reactions, relatively simple equipment, materials with porous morphology, and varied microstructure [89]. The microwave-assisted combustion makes the synthesis even faster, in the order of tenths of seconds (Figure 8).

The experimental setup for the MAC method can be obtained with a modified DMO. Nevertheless, attention to the safety issues must be thoroughly considered due to physical and chemical hazards involved in the combustion flames and toxic fumes. Moreover, microwave leakage from the modified setup should be avoided at all costs. An example of the synthesis configuration used for microwave-assisted combustion synthesis is depicted in Figure 8.

### 2.3. Microwave-Assisted Hydrothermal (MAH)

Hydrothermal synthesis refers to a crystallization technique of compounds under high-temperature aqueous solutions and at high vapor pressures. The crystal growth is performed in an apparatus consisting of a steel pressure vessel called an autoclave. A temperature gradient is maintained between the opposite ends of the chamber where the hotter end dissolves the ions. At the cooler end the crystals are formed. Advantages of the hydrothermal method include the ability to create crystalline phases which are not stable at the water boiling point.

The vessel barrier in the hydrothermal apparatus makes the synthesis in a resistive oven long and energetically inefficient. On the other hand, the microwave-assisted hydrothermal (MAH) method is an advantageous and powerful method to synthesize inorganic materials in a noticeably short time. The microwave radiation interacts directly with the water heating the solution in a short time. It makes the MAH synthesis highly efficient regarding energy conversion. The pressured solution allied to high temperatures allows crystallization of particles in the nanoscale as well. The vast number of compounds soluble in water, temperature modulation and pressure control allow researchers obtain nanoparticles with different composition, crystalline structures and morphologic shapes (rings, crystals, rods, spheres, cubes, urchin) [91].

**Figure 9:**
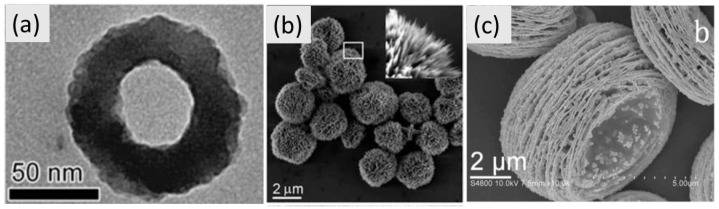
α-Fe_2_O_3_ nanorings (**a**), CuO nanourchins (**b**), and lamellar Cd(OH)_2_ (**c**) obtained by microwave-assisted hydrothermal method. Adapted from ref. [91]. Copyright © 2016, with permission from Elsevier.

Although there are examples of MAH synthesis using a modified DMO, it is advised to use a specialized setup due to the risk of explosion of the pressurized vessel [92]. Luckily, high-quality commercial setups are available. Among the advantages of MAH synthesis, the most surprising is obtaining nanoparticles with a narrow particle size distribution and varied morphologies (Figure 9). This is possible due to the homogeneous and fast dielectric heating by the microwave radiation.

### 2.4. Microwave-Assisted Sol-Gel (MASG)

Sol-gel synthesis combines two synthetical methodologies. In the first step, a polymeric 3D-framework is obtained by using organometallics or silane precursors. These precursors undergo hydrolysis and condensation process to form a gel, that contains evenly dispersed ions in the polymeric matrix. In a second step, to obtain the final material the gel precursor is thermally treated to remove carbon moieties. In the conventional sol-gel method, the heat treatment is performed using a resistive furnace. The MASG method utilizes microwave-assisted heating to thermally treat the gel precursors.

Birkel et al. [93]. obtained Pd-substituted LnFeO_3_ (Ln: Y, and La) using the MASG method. The prepared gel using citric acid and metal acetylacetonates was dried and then ground to obtain a powder that was subsequently heated in a microwave using the MASS setup. The noticeably short heating times in microwave reactions allowed the elucidation of the phase formation process showing that the reaction for forming LaFeO_3_ is completed after 90 s. In comparison with the furnace-based sol–gel or combustion methods, the MASG method has allowed the easy and reproducible tuning of material properties such as crystallite size and surface area, which are important parameters for photocatalytic activity.

Near-infrared (NIR) persistent luminescence nanoparticles are widely investigated for bioimaging and theranostics. However, if the particle’s hydrodynamic radius is bigger than 100 nm, the particles are sequestrated by the reticuloendothelial system (RES). Non-aqueous MASG synthesis can be an efficient route to obtain ultra-small nanoparticles (average size: 6 nm) of ZnGa_2_O_4_:Cr^3+^ persistent phosphors [94]. The ultra-small nanoparticle size reduces the RES clearance, enhances the blood circulation time, and allows widespread organ distribution. The non-aqueous benzyl alcohol route has been established as a powerful technique that takes place at a moderate temperature and pressure to obtain exceedingly small metal oxide nanoparticles with high crystallinity, purity, and reproducibility. The combination with the microwave heating offers a reduction of the reaction time (30 min) in comparison to traditional heating in an autoclave (48 h) [95]. One special advantage of this technique is that the one-pot process does not need further high temperature treatment, which generally leads to excessive particle growing. 

### 2.5. Other Preparation Methods

Coupled methodologies with microwave-assisted heating are an interesting alternative for synthesizing luminescent materials. Bi et al. [96] used a coupled microwave-ultrasound-assisted method to obtain luminescent Y_2_O_3_:Eu^3+^ materials. The method consists of the irradiation of a Y(NO_3_)_3_ water/ethanol solution using ultrasound (100 W) and microwave (150 W) for one hour. The method allows for nanoparticle formation (<100 nm) due to the local heating leading to fast crystallization and the ultrasound avoiding the excessive coalition of smaller particles. In this case, the obtained luminescent materials present high quantum yields of emission (*ca.* 80%).

The co-precipitation method consists of precipitate substances normally soluble in the precursor solution. In this method the mixing of the solution containing the soluble cations and anions forces the parts to interact, inducing nucleation and growth. After the particles surpass the critical mass the precipitation of the materials is observed. Usually aqueous solutions are used; however, it is possible to obtain precipitates in other polar solvents. Thongtem et al. [97] used a microwave-assisted co-precipitation method to obtain SrMO_4_ (M: W, and Mo) nanocrystals. The crystallization of the materials was performed by irradiating an ethylene glycol (EG) dispersion of the NaMO_4_ and Sr(NO_3_)_2_ salts. The effect of the EG solvent in the synthesis is not discussed, but the mildly chelating effect might play a role in retarding the precipitation and crystallization. Moreover, EG is an excellent microwave absorber, leading to a homogeneous heating profile. Due to the controlled crystallization in this method, they were able to produce small nanoparticles (~25 nm) with a narrow size distribution.

The comparison methods of main microwave-assisted methods is summarized in Table 3. 

## 3. Inorganic Luminescent Materials Obtained by Microwave-Assisted Methods

Inorganic luminescent materials can be obtained by various methods, such as conventional solid-state (CSS) synthesis, combustion synthesis, or sol–gel methods. However, the CSS method is the most widely used due to the combination of the easy processing and few steps that grants high reproducibility. In the CSS method, a high-temperature, direct reaction is expected where the solid reactants, with different grain sizes and randomly oriented surfaces, must react through ionic diffusion. The drawback of the solid-state reactions is the limited flux of diffusing species of precursors, impeding crystal growth and forming the final products [57]. The combination of low ionic diffusion with large temperature gradient during the resistive heating translates to long synthesis’ time at high temperatures (>1000 °C) to avoid inhomogeneous materials with poor optical properties. Thus, increasing the ionic diffusion and reducing the temperature gradient can be an alternative method of obtaining efficient inorganic luminescent materials swiftly. In the following sections, we review several classes of luminescent materials obtained by microwave-assisted methodologies.

### 3.1. Oxides

Several luminescent materials are based on oxides doped with activator ions, e.g., Y_2_O_3_:Eu^3+^ [96], Lu_2_O_3_:Tb^3+^ [101]. Usually, oxide luminescent materials are prepared from oxide precursors in a solid-state synthesis. In addition, carbonate precursors are commonly used with the same intent to obtain the respective oxide after the decomposition of carbon moieties at moderate temperatures. Since most of the metal oxides are low-lossy materials, having considerable penetration depth, they act as microwave-transparent materials. Thus, hybrid heating is used to achieve the needed high temperature for the synthesis.

**Figure 10:**
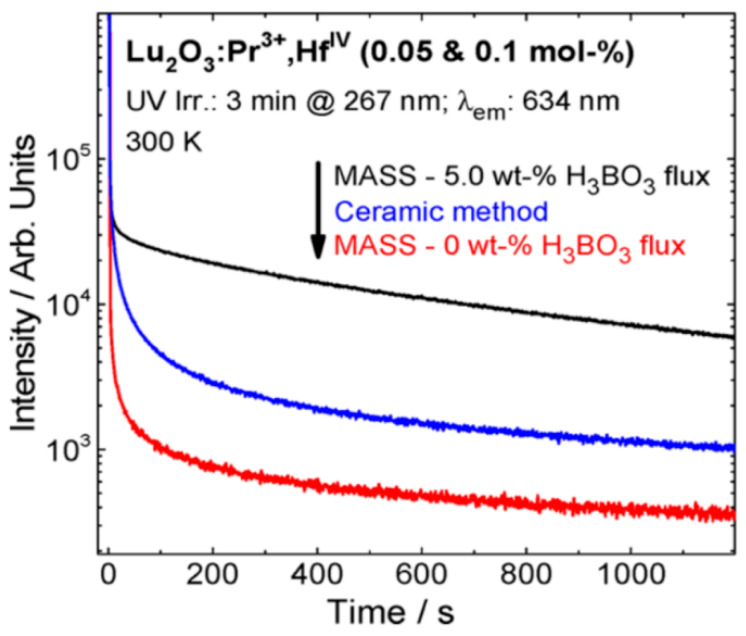
Persistent luminescence decay curves of Lu_2_O_3_:Pr^3+^, Hf^4+^ materials obtained by conventional (blue curve), and microwave-assisted (black curve) solid-state methods, and the PeL decay of the MASS obtained materials without H_3_BO_3_ flux (red curve) [62]. Copyright © 2016, American Chemical Society.

Pedroso et al. [62] used the MASS method to obtain the Lu_2_O_3_:RE^3+^ (RE: Pr, Eu, and Tb) persistent luminescent materials using a modified DMO setup. Rare-earth doped lutetia is a known family of materials with long persistent luminescence decay, especially the Lu_2_O_3_:Tb^3+^, Ca^2+^ [82]. Lu_2_O_3_-based materials require rather extreme preparation conditions in solid-state synthesis needing high temperature (~1700 °C) in high vacuum or H_2_-N_2_ gas flow [102]. When the MASS method is employed, the material can be prepared in one step of 22 min. Despite the short synthesis time, the materials are highly crystalline and have large storage capacity compared to the materials prepared by the conventional solid-state method (Figure 10). Most importantly, complete reduction of Tb_4_O_7_ to Tb^3+^ was achieved by the reducing atmosphere generated in-situ during the synthesis.

### 3.2. Aluminates

Aluminates are commonly used as a host for LED and energy storage phosphors. The benchmark persistent luminescence material is a doped strontium aluminate with the general formula SrAl_2_O_4_:Eu^2+^, Dy^3+^ [103] that has the best efficiency to date. Interestingly, the canonical material for pc-LEDs is also an aluminate, Y_3_Al_5_O_12_:Ce^3+^ [104]. In 2017, Finley et al. [105] used microwave-assisted heating to synthesize SrAl_2_O_4_:Eu^2+^, Dy^3+^ phosphors starting from reverse micelle precursor. The reverse micelle was prepared in an aqueous metal nitrates solution with a combination of components for emulsion (CTAB, n-Heptane, and 1-butanol). The prepared reverse micelle was submitted to a hybrid microwave heating using activated carbon as a susceptor. The total irradiation time was 14 min, yielding nearly phase-pure materials. The combined reverse micelle with microwave-assisted heating produced particle sizes 70% smaller (d_0.5_ = 4.2 μm) than the conventional MASS (d_0.5_ = 14.3 μm). Despite the similar photoluminescence properties, the reverse micelle material has a higher temperature quenching and slightly larger storage capacity.

**Figure 11:**
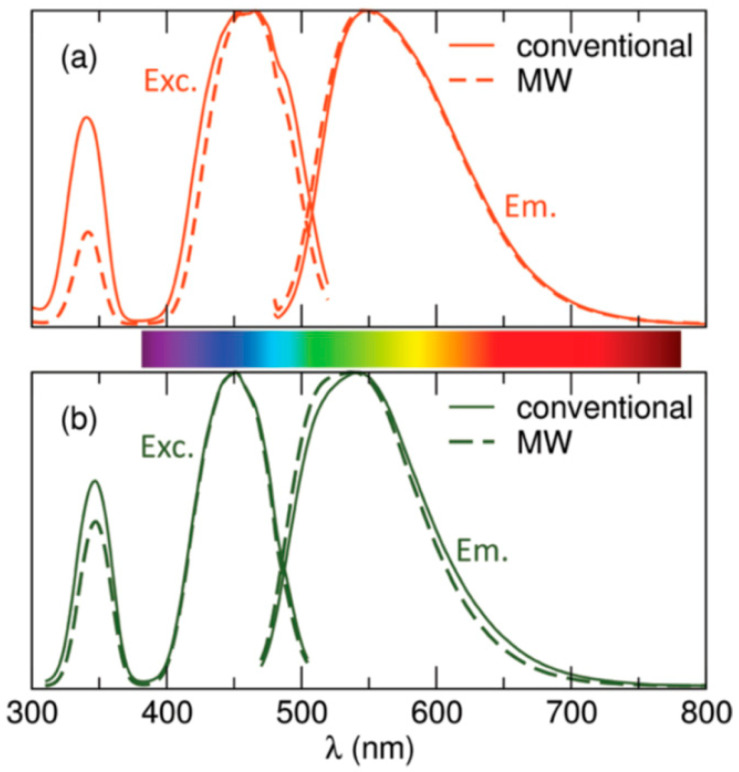
Excitation and emission spectra of Y_3_Al_5_O_12_:Ce^3+^ (**a**) and Lu_3_Al_5_O_12_:Ce^3+^ (**b**) obtained by both conventional (line) and MASS (dashed line) methods [83]. Copyright © 2012, American Chemical Society.

In 2012, Birkel et al. [83] used the MASS method to prepare RE_3_Al_5_O_12_:Ce^3+^ (RE: Y, and Lu) starting from RE_2_O_3_ and Al_2_O_3_, and small quantities of fluoride fluxes. The synthesis is efficient for obtaining highly crystalline garnet phosphors with similar quantum yields to conventional solid-state samples. Usually, luminescent rare earth aluminum garnets need thermal treatment at high temperatures (1500 °C) for several hours to form the material. On the other hand, the MASS synthesis provided an energy saving of 99% with nearly identical optical and structural quality (Figure 11). The quantum yields for the MASS-obtained aluminum garnets were 88% and particle sizes were slightly smaller.

### 3.3. Silicates

Silicate is an essential class of inorganic host matrices for luminescent materials. The most famous example is the efficient blue-emitting Sr_2_MgSi_2_O_7_:Eu^2+^ phosphor.

**Figure 12:**
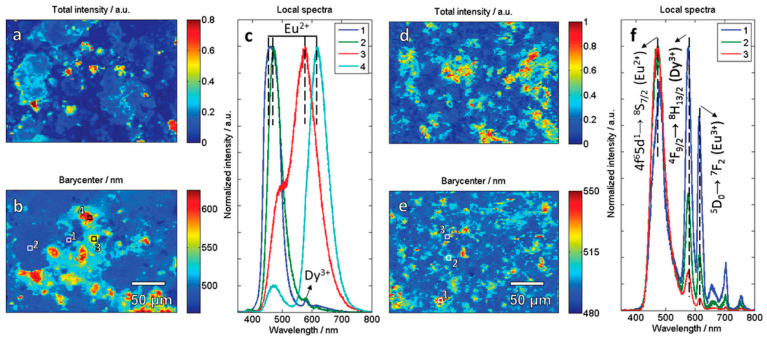
SEM-CL hyperspectral images of the Sr_2_MgSi_2_O_7_:Eu^2+^(1%), Dy^3+^(1%) materials obtained by CSS (**a**,**b**) and MASS (**d**,**e**) method. The numbers in the images (**b**,**e**) correspond to the area of the local spectra extracted for the CSS (**c**) and MASS (**f**) materials. Reproduced from Ref. [57] with permission from The Royal Society of Chemistry.

In 2020, Carvalho et al. [57] studied the MASS synthesis of Sr_2_MgSi_2_O_7_:Eu^2+^, Dy^3+^ persistent phosphors and microwave irradiation’s effects on the homogeneity and storage capacity. Comparing the conventional and microwave-assisted methods on obtaining the Sr_2_MgSi_2_O_7_:Eu^2+^, Dy^3+^ shows that the MASS method provides higher dopant homogeneity in the crystal due to increased ionic diffusion induced by microwave irradiation. However, the local atmosphere generated by carbon susceptor is sometimes inefficient to reduce the activator ions. In this case, decoupled, two-step synthesis is beneficial to obtain Sr_2_MgSi_2_O_7_:Eu^2+^, Dy^3+^ materials with high quantum yields, and large storage capacities. In the first step, highly homogeneous materials are obtained due to the increased diffusion in MASS synthesis (Figure 12). The second step reduces the activator ions in mild temperatures achieving optimum Eu^2+^ concentrations for high-brightness and a large storage capacity.

The MASS method can also be beneficial when the synthesis’s precursor is volatile or decomposes at high temperatures. The swift nature of the dielectric heating allows the reaction to occur before evaporation or degradation takes place. Brgoch et al. [106] obtained Ce^3+^-doped silicates with complex composition, Na_3_YSi_2_O_7_, using a MASS methodology. The rapid MASS method allows for synthesizing the materials using stoichiometric amounts of the precursors, unlike conventional preparations that must account for sodium carbonate’s evaporation.

### 3.4. Aluminosilicate

Aluminosilicate consists in AlO_4_ and SiO_4_ units interconnected in a 3D framework. Famous example are the zeolites and minerals derived from the sodalite structure. Hackmanite, a sulfide containing sodalite, is a material that exhibits persistent luminescence when doped with Ti ions [107,108]. The white emission has long durations and is the record-long for non-rare earth persistent phosphors [109,110]. The complex structure is usually synthesized using a two-step solid-state synthesis that takes ca. 50 h combined. In 2019, the series of sodalite (Na,M)_8_Al_6_Si_6_O_24_(Cl,S)_2_ (M: Li, K, Rb) materials were synthesized using a zeolite precursor applying the MASS method [111]. The structural similarities of the precursors and products, together with the enhanced ionic diffusion induced by MW radiation, reduce the synthesis’ time to 18-20 min. The formation of the material goes through a crystalline intermediate, NaAlSiO_4_, showing a sharp transition from zeolite to sodalite after 8 min of MW irradiation.

**Figure 13:**
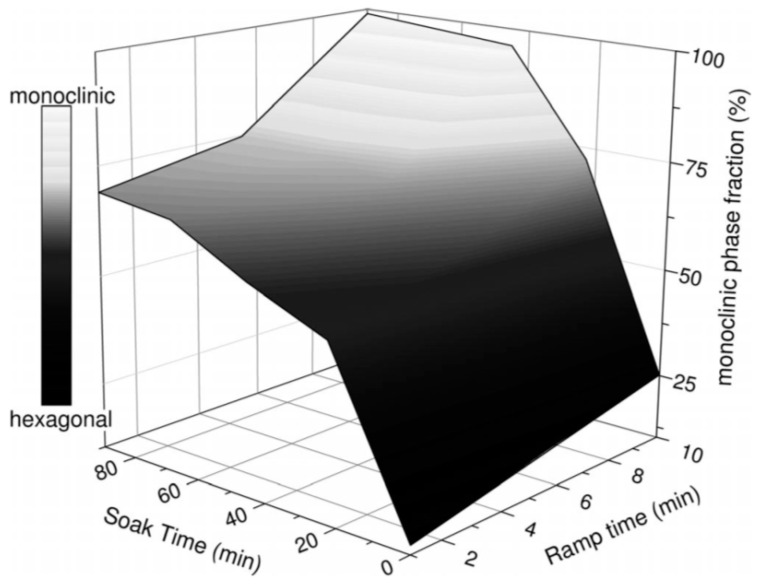
Contour plot of the conversion from the hexagonal phase to the monoclinic phase of Eu-doped (Ba_1-x_Sr_x_)Al_2_Si_2_O_8_ materials. The ramp time and soak time (in minutes) of the microwave reaction are presented on the x and y axes, respectively, while the refined phase fractions are presented on the z axis [112]. Copyright © 2014, with permission from John Wiley and Sons.

Brgoch et al. studied the MASS synthesis of the Eu-doped (Ba_1-x_Sr_x_)Al_2_Si_2_O_8_ aluminosilicate materials. The method yields nearly phase-pure materials, which are often difficult to achieve using conventional solid-state methods. Both of the phases, hexagonal and monoclinic, can be synthesized with this method by varying the irradiation time and power levels. Short irradiation (2 min) is enough to synthesize the hexagonal phase, whereas longer times (100 min) lead to the monoclinic phase (Figure 13). The order of the crystallization was interpreted using the Ostwald law wherein the less stable polymorph crystallizes first.

### 3.5. Sulfides and Oxysulfides

Sulfides and oxysulfides inorganic materials are commonly used as host matrices for luminescent materials. The presence of sulfur, which is bigger and more polarizable compared to oxygen, makes these hosts interesting for emission color tuning of crystal field-sensitive activators, such as Eu^2+^ and Ce^3+^.

The synthesis of alkaline earth sulfides is exceedingly complex, usually needing special synthesis setups. For instance, a reducing atmosphere composed by toxic H_2_S gas is often needed. The usage of poisonous gas limits the synthesis to specialized furnaces. Moreover, the synthesis of the sulfides by conventional method must consider the evaporation of the sulfur during the process. In 2020, Santos et al. [73] synthesized SrS materials doped with various rare-earth ions to compare the co-dopant effect in PeL storage capacity. The MASS synthesis was applied successfully to obtain SrS materials in a single step without using H_2_S to reduce the sulfur to sulfide anions. Although there were small SrSO_4_ impurities, crystalline rare-earth-doped SrS materials were obtained with the facile method in 22 min. The decay time of the persistent luminescence was measured in luminance mode, indicating overall good performance of the PeL phosphors.

**Figure 14:**
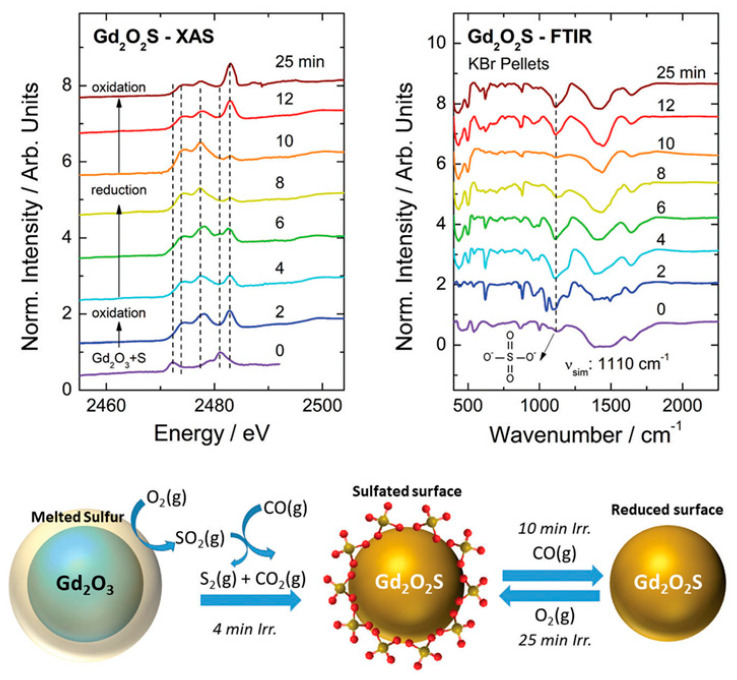
SR-XANES of the sulfur K-edge (**top left**) and FTIR spectra (**top right**) of the Gd_2_O_2_S materials obtained by the MASS method for different durations of microwave exposure. Schematic representation of oxysulfide formation with the participation of the gases assisting the redox process of sulfur (**bottom**). Reproduced from Ref. [76] with permission from The Royal Society of Chemistry.

Sulfur-containing materials are prone to oxidation depending on the synthesis conditions. For instance, La_2_O_2_S materials suffer oxidation to La_2_OSO_4_ at high temperatures in an oxygen-containing atmosphere [113]. Thus, the synthesis of sulfur-containing materials using MASS synthesis must be finely controlled to avoid excessive temperature rise. In 2018, Carvalho et al. [76] used the MASS method to obtain the RE_2_O_2_S (RE: La, Gd, and Y) materials doped with Ti^3+/4+^ and Mg^2+^ ions. After the 25 min irradiation, the crystalline phase is mainly the RE_2_O_2_S hexagonal phase. The analysis of the sulfur K-edge XANES and FTIR spectra showed rapid oxidation of the elemental sulfur (S^0^) to sulfate (S^6+^) in the first 2 min. It is shown that the reduction takes place from 2 to 10 min, where only S^2-^ species are observed (Figure 14). The increased irradiation time promotes partial oxidation of S^2-^ to SO_4_^2-^ species. The results showed a great versatility of the MASS synthesis of sulfur-containing materials where different irradiation times allied with the rapid heating and cooling rates can lead to different sulfur valences.

### 3.6. Oxometalates

Metalates are the name given to complex anions composed of a metal bonded to a different type of anions. In the case of the oxometallate, a transition metal is bonded to oxygen atoms to form complex anions in various forms, e.g., MnO_4_^−^, VO_4_^3−^, CrO_4_^2−^, WO_4_^2−^, and MoO_4_^2−^.

Rare earth-doped tungstate and molybdate matrices are extensively studied as luminescent materials, and are usually obtained by solution-phase methods, such as co-precipitation, sol-gel, hydrothermal, and Pechini. These methods generally require more than one step to separate the material from the solution or heat treating the materials to achieve phase-pure phosphors. Several scheelites have been prepared by using the microwave-assisted solution-phase methods, such as NaLa(MoO_4_)_2_ [114], Na(Ca,Sr)(La,Gd)(MoO_4_)_3_ [115,116], and (Sr,Pb)(La,Gd,Y)_2_(MoO_4_)_4_ [117]. Solid-state synthesis using microwave heating of oxometallates is scarce, with BaMoO_4_ [118] and CdWO_4_ [119] being some examples.

In 2018, Zhai et al. [120] prepared the CaMoO_4_:Eu^3+^, Dy^3+^ scheelite using the MASS method with carbon as susceptor and metal carbonates as precursors. The irradiation program was 30 min at 560 W power level, using a DMO. The synthesized materials possess interesting morphology where micrometric cubes are formed by close-packed quasi-spherical grains.

**Figure 15:**
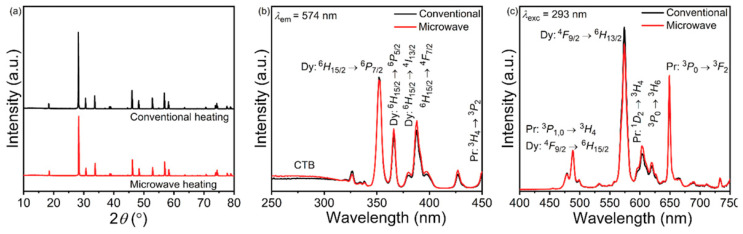
(**a**) XRD patterns, (**b**) excitation spectra, and (**c**) emission spectra of quinary molybdate phosphors NaLa_0.95_Pr_0.025_Dy_0.025_(MoO_4_)_2_ synthesized using conventional and microwave heating. Reproduced from ref. [121] with permission from The Royal Society of Chemistry.

In 2020, Perera et al. [121] described the MASS synthesis of a series of NaRE(MO_4_)_2_ materials (RE: La, Pr, Eu, Dy; M: Mo, W). The synthesis was conducted using pure oxide precursors using various irradiation programs. The total time of the synthesis varied in the interval of 6 to 27 min. The authors reported that the synthesis was robust, fast, and that it was simple to obtain structurally complex metallates. As for the luminescence properties, the materials obtained by MASS synthesis were compared to those from the conventional heating method, and the light output was nearly identical (Figure 15), apart from NaLa_0.95_Eu_0.05_(MoO_4_)_2_ material that exhibits higher emission intensity for MASS-obtained material.

In 2015, Duée et al. used the microwave-assisted hydrothermal method to obtain luminescent nanovanadates with the formula Y_1-x_Eu_x_VO_4_. The method yields small nanoparticles (~10–15 nm) in only 15 min of microwave irradiation. The materials were reported as having efficiencies higher than the state-of-the-art inorganic optical material in liquid phase detection of H_2_O_2_.

### 3.7. Other Materials

Du et al. [122] used the MASS method to obtain a novel Mn^4+^-doped double-perovskite germanate structure with the formula La_2_MgGeO_6_. The approach consisted of two steps, first irradiating the sample with microwaves for 40 min, and then conventional thermal treatment for 3 h. Mn^4+^ phosphors are emerging as a new class of efficient NIR emitters for bioimaging and night-vision surveillance. However, these materials’ preparation can be complex as the manganese ions can assume different valence states. This approach was used because the hybrid heating using charcoal generates CO gas, which acts as a reductant of Mn^4+^ to Mn^2+^, at least partly. The reduction is undesirable because it changes the optical performance and even kills the emission ultimately. The second step of mild temperature heating allows the oxidation of the Mn ions to the tetravalent form. Combining these two effects, it is possible to obtain a highly dispersed dopant, homogeneous crystal structure, and optimized luminescence performance.

Several classes of materials were prepared by microwave-assisted methodologies, and the selected examples are presented in Table 4. The description of the methods, microwave program, and susceptor can be used as a guide to plan a synthetic route using microwave-assisted methodologies.

## 4. Conclusions and Outlook

In conclusion, the future of luminescent materials obtained by microwave methodologies is bright yet challenging. The microwave-assisted synthesis methodologies are incredibly versatile and are proved to reduce energy consumption and considerably lower the synthesis time of inorganic luminescent materials. Not only is this method more cost-effective than its conventional heating counterpart, but it also has an additional effect on the synthesis, leading to increased reaction rates due to the enhanced ionic diffusion promoted by the electromagnetic field of the MW. Dielectric heating is also an excellent strategy to obtain homogeneous luminescent materials, ideal for illumination purposes, once the intense local heat generation leads to a slight temperature gradient. The rapid heating and quenching of microwave-assisted heating can induce formation of structural defects that allow for high storage capacity of the persistent phosphors.

The shortcoming of the microwave methodologies is sometimes the lack of quantitative comparison between the microwave-obtained materials and other methods, making the advantages challenging to interpret. Furthermore, the methodologies, especially the solid-state one, need to be consolidated in the luminescence community as a recognized method for obtaining phosphor materials. If the radiation hazards are appropriately controlled, novel customized setups can be designed for other methods, such as reflux synthesis using organic solvents and ionic liquids, that might allow obtaining nanoparticles with controlled morphology, such as NaYF_4_. The design of a dedicated microwave irradiation reactor cavity could also open new avenues for luminescent materials, leading to possibilities of temperature and atmosphere control.

The combination of these properties makes microwave-assisted synthesis an excellent method to obtain luminescent materials that benefit from high crystallinity, well-dispersed dopants, and homogeneous structures. These properties translate to high quantum yields, high brightness, and large storage capacity. Moreover, the microwave-assisted methods allow for fast screening for compositional tailoring, a widespread strategy when tuning the color emission of crystal field-sensitive ions, such as Eu^2+^ and Ce^3+^.

## Figures and Tables

**Table 1:** Microwave dielectric properties (tan δe and dp) and average heating rate of selected susceptor materials. The values are from the references [76,79,80,81].

Material	tan δ_e_	dp (m)	Average Heating Rate (°C/s)
Amorphous Carbon	-	-	21
Graphite powder	0.36–0.67	0.013–0.002	10
Activated Carbon	0.31–0.9	0.007–0.03	4.8
Fe_3_O_4_ (Magnetite)	0.02	-	7.6
CuO	0.087	0.21	2.7
SiC	0.388	0.019	0.3–2.8
Co_2_O_3_	0.0002	-	7.2
NiO	0.001	-	3.5

**Table 2:** Dielectric tangent loss (tan δe) of various solvents. Values were obtained at 20 °C and 2.45 GHz microwave frequency [56].

Solvents	tan δ_e_
Water	0.15
Ethylene glycol	1.35
Ethanol	0.941
Methanol	0.659
DMSO	0.825
1-Butanol	0.571
DMF	0.161
Acetic Acid	0.174
Dichloromethane	0.042

**Table 3:** Comparison of microwave-assisted methodologies used to obtain luminescent inorganic materials.

Method *	Advantages	Disadvantages	Benefits for Luminescent Materials	Examples
MASS	Energy saving Fast processing times (minutes) Increased reaction rates Increased ionic diffusion High homogeneity	Few commercial setups available DMOs can struggle with the reproducibility In modified DMOs safety issues must be addressed	High QY High storage capacity Increased lifetimes Less prone to concentration quenching	Y_3_Al_5_O_12_:Ce^3+^ [83] Sr_2_MgSi_2_O_7_:Eu^2+^, Dy^3+^ [57]
MAC	Fast processing times (seconds) Porous materials Reduced particle sizes	Morphology control is difficult Carbon residues In modified DMOs safety issues must be carefully addressed	Combination of high homogeneity and moderately small particle sizes	Y_3_Al_5_O_12_:Er^3+^, Yb^3+^ [85] Gd_2_O_3_:Er^3+^, Yb^3+^, Zn^2+^ [90]
MASG	Fast processing times (minutes) Nanoparticulate materials Morphology control	Carbon residues Expensive and reactive precursors	Nanoparticulate materials Possible to use as a coating method	ZnGa_2_O_4_:Cr^3+^ [94] M_2_SiO_4_:Eu^2+^ (M:Ca,Ba) [98]
MAH	Fast processing times (Seconds to minutes) Nanoparticulate materials Finely controlled morphology	DMOs cannot be used due to safety issues Expensive commercial setups	Nanoparticles for increased applicability in bioimaging and photodynamic therapy	YVO_4_:Eu^3+^ [99] ZnWO_4_:Dy^3+^ [100]

* Microwave-assisted solid-state (MASS), combustion (MAC), sol-gel (MASG), and hydrothermal (MAH).

**Table 4:** Inorganic hosts for luminescent materials obtained by microwave-assisted methodologies.

Class	Composition	Method	Susceptor	MW Program	Ref.
Aluminate	BaMgAl_10_O_17_:Eu^2+^	MAC	Urea and Water	15 min @ 850 W	[123]
	Ca_3_Al_2_O_6_:Eu^3+^	MAC	Solution	Until Ignition @ 560 W	[124]
	Lu_3_Al_5_O_12_:Ce^3+^	MASS	Carbon	18–25 min@850–1300 W	[83]
	MgAl_2_O_4_	MASS	Carbon	100 min@700 W	[75]
	SrAl_2_O_4_:Eu^2+^, Dy^3+^	MASS	Carbon	9 min@960 W + 5 min @480 W	[105]
	Sr_3_Al_2_O_6_:Eu^2+^, Dy^3+^	MASS	Carbon	20 min @ 650 °C	[125]
	Y_3_Al_5_O_12_:Ce^3+^	MASS	Carbon	18–25 min@850–1300 W	[83]
	Y_3_Al_5_O_12_:Ce^3+^	MAC	Solution	5,10,15 min@900 W	[126]
	Y_3_Al_5_O_12_:Er^3+^, Yb^3+^	MAC	Solution	Not provided	[85]
	ZnAl_2_O_4_:Cu^+^	MAC	Solution	1 min@900 W	[127]
Aluminosilicate	Al_6_Si_2_O_13_:Eu^3+^	MASG	Solution	30–45min@680–800 W	[128]
	(Ba_1-x_Sr_x_)_1-y_Eu_y_Al_2_Si_2_O_8_	MASS	Carbon	10 min@1000 W + 30–90 min@375 W	[112]
	(Na,M)_8_Al_6_Si_6_O_24_(Cl,S)_2_	MASS	Carbon	18 min@450 W	[111]
Fluoride	KMF_3_ (M = Zn, Mg, Mn, Co)	MASS	None	10 min@1100 W	[129]
	NaYF_4_:Yb^3+^,R^3+^ (R^3+^: Er, Tm, Yb)	MAH	Solution	5–10 min@60–180 °C	[130]
	KYF_4_:R^3+^ (R: Ce, Tb)	MAH	Solution	4 h@200 °C	[131]
	LiYF_4_:R^3+^ (R: Eu, Tb, Dy)	MAH	Solution	15 min@140 °C	[44]
Germanate	La_2_MgGeO_6_:Mn^4+^	MASS	Carbon	40 min@1000 W	[122]
	Zn_2_GeO_4_:Mn^2+^,Yb^3+^	MAH	Solution	Not provided	[132]
Molybdate	CaMoO_4_:Eu^3+^,Dy^3+^	MASS	Carbon	30 min@560 W	[120]
	CaGd_2_(MoO_4_)_4_:Ho^3+^,Yb^3+^	MASG	Solution	30 min@1250 W	[133]
	CaMoO_4_:Er^3+^, Yb^3+^	MAC	Ethylene Glycol	23 min@1250 W	[134]
	CdMoO_4_:Eu^3+^	MAH	Solution	10 min @ 180 °C	[135]
	Cs_5_Bi(MoO_4_)_4_	MASS		10min@850 W	[136]
	Na_2_MoO_4_	MASS	Carbon	3 min@840 W (9 cycles)	[121]
	NaY(MoO_4_)_2_:Yb^3+^, Tm^3+^	MAH	Solution	1 h@180 °C	[137]
	NaCaGd(MoO_4_)_3_:Ho^3+^, Yb^3+^	MASG	Solution	30 min@1250 W	[138]
	NaCaLa(MoO_4_)_3_	MASG	Solution	30min@1250 W	[139]
	NaCaGd(MoO_4_)_3_:Er^3+^, Yb^3+^	MASG	Solution	30 min@1250 W	[140]
	NaY(MoO_4_)_2_:Eu^3+^	MAH	Solution	10 min@170 °C	[141]
	NaEu(MoO_4_)_2_	MASS	Carbon	3 min@960 W (6 cycles)	[121]
	NaPbLa(MoO_4_)_3_:Er^3+^, Yb^3+^	MASG	Solution	30 min @ 1250 W	[117]
	PbGd_2_(MoO_4_)_4_:Er^3+^, Yb^3+^	MASG	Solution	30 min@1250 W	[142]
	NaLa_0.95_Eu_0.05_(MoO_4_)_2_	MASS	Carbon	3 min@960 W (9 cycles)	[121]
	NaLa_0.95_Pr_0.025_Dy_0.025_(MoO_4_)_2_	MASS	Carbon	3 min@960 W (6 cycles)	[121]
	NaSrLa(MoO_4_)_3_:Er^3+^, Yb^3+^	MASG	Solution	30 min @ 1250 W	[116]
	SrMoO_4_	MAP	Ethylene Glycol	20 min@180 W	[97]
Nitride	Ca_2_Si_5_N_8_:Eu^2+^	MASS	None	10 min@100–600 W	[143]
Oxide	Lu_2_O_3_:R^3+^ (R:Pr, Eu, Tb)	MASS	Carbon	12 min@1000 W + 10 min@900 W	[62]
	Y_2_O_3_:Eu^3+^	US—MA	Water	60 min@150 W	[96]
	SnO_2_:Eu^3+^	MAC	Ethylene Glycol	Not Provided	[144]
	TiO_2_:Sm^3+^	MAH	Solution	31 min @ 850 W	[145]
	HfO_2_:Eu^3+^	MAH	Solution	20 min @ 260 °C	[146]
Oxysulfide	R_2_O_2_S:Ti,Mg (R:La, Gd, Y)	MASS	Carbon	10 min@900 W + 15 min@800 W	[76]
	Gd_2_O_2_S:Eu^3+^	MASS	Carbon	2 min@167, 500, and 1000 W	[147]
	Gd_2_O_2_S:Tb^3+^	MASS	Carbon	10 min@900 W + 15 min@800 W	[77]
Phosphate	LaPO_4_:Ce^3+^, Tb^3+^	MAP	Ionic Liquid	10 s@800 W	[148]
	LaPO_4_:Eu^3+^, Li^+^	MASG	Gel	30 min@80 °C + 30 min@800 °C	[149]
	CePO_4_:Tb^3+^	MAP	Solution	4 min@700 W	[150]
	Ca_3_(PO_4_)_2_:Gd^3+^, Pr^3+^	MAH	Solution	1 h @ 150 °C (300 W)	[151]
	YPO_4_:R^3+^(R:Eu, Ce, Tb)	MAH	Solution	Not provided	[152]
	Sr_2_P_2_O_7_:Ce^3+^, Tb^3+^	MASS	Solution	15 min @ 1000 °C	[153]
Silicate	Bi_4_Si_3_O_12_:Eu^3+^	MASS	Not provided	15 min @ 750 °C	[154]
	Ca_2_MgSi_2_O_7_:Eu^2+^	MASS	Carbon	Not provided	[155]
	M_2_SiO_4_:Eu^2+^ (M: Ca, Ba)	MASG	Carbon	10 min + 12 min @ 1000 W	[98]
	Mg_2_SiO_4_:R^3+^ (R: Eu, Tb)	MASG	Solution	12 min @ 800 W	[156]
	Na_3_YSi_2_O_7_:Ce^3+^	MASS	Carbon	8 min@625 W + 15 min@375 W	[106]
	Sr_2_SiO_4_:Tb^3+^	MASG	Solution	14 min @ 800 W	[157]
	Sr_2_MgSi_2_O_7_:Eu^2+^,Dy^3+^	MASS	Carbon	12 min@1000 W + 10 min@900 W	[57]
	Sr_3_SiO_5_:Eu^2+^	MASS	Carbon	20 min @3200 W	[158]
Sulfide	ZnS:Mn	MAH	Solution	10 min@800 W	[159]
	Zn_1-x_Cd_x_S	MAC	Solution	Not provided	[160]
	ZnS:Pb^2+^	MASS	None	4 h@ 700 °C	[84]
	SrS:Eu^2+^,R^3+^ (R: Ce, Sm, Er, Dy)	MASS	Carbon	12 min@1000 W + 10 min@900 W	[73]
Titanate	CaTiO_3_:Eu^3+^	MASS	Carbon	10–60 min@528–800 W	[161]
	Lu_2_Ti_2_O_7_:Yb^3+^, Er^3+^	MAH	Solution	1 h@200 °C (100 W)	[162]
	BaTiO_3_	MASS	None	1–5 min@2000 W	[163]
Tungstate	CaGd_2_(WO_4_)_4_:Er^3+^, Yb^3+^	MASG	Solution	30 min @ 1250 W	[164]
	Li_3_BaSr(La_1-x_Eu_x_)_3_(WO_4_)_8_:Eu^3+^	MASS	Not provided	20 min@600 °C + 10 min@900 °C	[165]
	Li_2_Gd_4_(WO_4_)_7_	MASS	Carbon	10 min@800 °C	[166]
	NaGd(WO_4_)_2_:Yb^3+^, Tm^3+^, Ho^3+^	MAH	Solution	2 h @ 200 °C	[167]
	ZnWO_4_:Dy^3+^	MAH	Solution	1 h@140 °C	[100]
	Na_2_WO_4_	MASS	Carbon	2 min@1080 W (3 cycles)	[121]
	NaEu(WO_4_)_2_	MASS	Carbon	3 min@1080 W (6 cycles)	[121]
	NaLa_0.95_Eu_0.05_(WO_4_)_2_	MASS	Carbon	3 min@1080 W (9 cycles)	[121]
	NaLa_0.95_Pr_0.025_Dy_0.025_(WO_4_)_2_	MASS	Carbon	3 min@1080 W (9 cycles)	[121]
	NaY(WO_4_)_2_: Ho^3+^, Yb^3+^	MAH	Solution	2 h @ 180 °C	[168]
	NaLaMgWO_6_:Dy^3+^, Tm^3+^	MASS	None	10 min @ 900 °C	[169]
Vanadate	CeVO_4_	MAH	solution	10 min@600 W	[170]
	EuVO_4_	MAH	Solution	15 min@150 °C	[99]
	Y_0.5_Eu_0.5_VO_4_	MAH	Solution	15 min@150 °C	[99]
	YP_x_V_1-x_O_4_:R^3+^ (R: Eu, Dy, Sm)	MAH	Solution	30 min @ 150 °C	[171]
	YVO_4_:R^3+^ (R: Eu, Dy, Sm)	MAH	Solution	20 min @ 270 W	[172]
